# Improving Safety, Efficiency, Cost, and Satisfaction Across a Musculoskeletal Pathway Using the Digital Assessment Routing Tool for Triage: Quality Improvement Study

**DOI:** 10.2196/67269

**Published:** 2025-04-25

**Authors:** Cabella Lowe, Laura Atherton, Peter Lloyd, Anna Waters, Dylan Morrissey

**Affiliations:** 1 Centre for Sports and Exercise Medicine William Harvey Research Institute Queen Mary University of London London United Kingdom; 2 Joint Health Hub Ormskirk & District General Hospital Mersey & West Lancashire Teaching Hospitals NHS Trust Ormskirk United Kingdom; 3 Optima Health Sheffield United Kingdom; 4 Department of Physiotherapy Barts Health NHS Trust London United Kingdom

**Keywords:** triage, musculoskeletal, quality, safety, effectiveness, improvement, outcomes, cost, value

## Abstract

**Background:**

Musculoskeletal (MSK) conditions are prevalent and increasing in Western-style economies, associated with an aging population and reduced physical activity levels. Prevention, early detection, and treatment can enable people to live in good health, remain independent and socially connected, and have economic advantages for society, such as reducing pressure on health and social care services. Triaging patients safely and effectively to the right care, for the first time improves outcomes and reduces costs, with digital solutions offering potential advantages over traditional methods.

**Objective:**

We evaluated the impact of introducing a digital assessment routing tool (DART) on safety, efficiency, cost, and satisfaction across a National Health Service (NHS) England MSK service.

**Methods:**

We designed a quality improvement study using a Plan-Do-Study-Act design and Integrated Knowledge Translation model, with DART as the first point of contact for self-referring patients with MSK conditions. Patients completed a web-based DART assessment independently, or with administrative telephone support. The primary safety outcome was measured by agreement between clinician judgment and safety incident surveillance. Quantitative and qualitative methods were used to measure secondary outcomes of efficiency, cost, and satisfaction. Analysis was completed collaboratively between researchers and the NHS service team with reference to 4 months of prestudy data. Three consecutive study cycles were completed over a 4-month period between February and May 2024 with 4076 self-referring patients between the ages of 16-104 (mean 59) years.

**Results:**

Ninety-three percent of patients self-assessed using DART with the remainder assisted by an administrator. All predefined outcome targets were met for all measures. Agreement between clinicians and DART was 96%, no safety incidents occurred, there was immediate stratification of 401 (9.8%) urgent cases, and 203 fewer cases requiring clinical escalation following initial clinician contact. Administrative time to process self-referrals was reduced by 51% with a cost saving of £80.16 (US $101.30) per 100 referrals. Introduction of a new route to self-management for less complex conditions showed a cost reduction per patient of 73%, giving a saving of £1272.90 (US $1605.56) for 100 referrals. Routing to a new osteoarthritis knee program would reduce costs for these patients by 63%, equating to £220.35 (US $278.46), if implemented. Further potential savings of £28,476 (US $37,320)/annum could be realized using DART to screen for service eligibility criteria. Patient satisfaction was consistent throughout the study, with a mean of 90%. Service administrators and clinicians rated the new process as a positive service improvement.

**Conclusions:**

The introduction of DART demonstrated positive outcomes in all measures and presented opportunities to improve safety and efficiency, reduce cost, and improve patient and clinician satisfaction across an NHS MSK pathway. In addition, the successful delivery of an Integrated Knowledge Translation Approach showed the benefits of collaborative working between researchers, clinicians, and other service staff.

## Introduction

The following Quality Improvement Study was designed, conducted, and reported in accordance with the Standards for Quality Improvement Reporting Excellence Framework (SQUIRE 2.0) [1].

### Background

Musculoskeletal (MSK) conditions are prevalent and increasing, associated with an aging population and reducing levels of physical activity in Western-style economies [2,3]. They pose a financial and societal challenge, with the cost to the UK National Health Service (NHS) estimated at £6.3 billion (US $8.26 billion) in 2022-2023 [4].

Prevention, early detection, and treatment can enable people to live in good health, remain independent and socially connected, as well as have economic advantages for society, such as reducing the pressure on health and social care services and reducing costs as a result of people being unable to work [5].

MSK services in the United Kingdom were not realizing their potential even prior to the COVID-19 pandemic, due to increasing demand and workforce supply issues. This has led to lengthy and increasing waiting times, delays in diagnosis, and people with complex conditions often cycling between services without getting the support they need [6]. The pandemic prevented all but urgent and emergency cases from being seen, resulting in the current unprecedented waiting list backlogs [7]. Furthermore, a national rise in the complexity of presentation of cases of MSK conditions has been noted [4], producing increased demands on clinician resources, particularly less experienced staff.

“Getting It Right First Time” by stratifying patients to the correct level of intervention at the first point of contact is considered key in ensuring patient safety and improving outcomes of MSK conditions and efficiency across the MSK pathway, with early identification of patients requiring urgent medical review considered a priority [5,6]. For this reason, patient safety was the primary measure for our study.

Remote physiotherapist-led MSK triage is widely used to stratify patients to the correct level of care, and has proven effective in reducing waiting times, unwarranted variation in clinical pathways, clinician caseload, and cost [6-8]. However, the principal rate-limiting factor in delivering triage is the availability of staff [9]. It has been suggested mobile health technology could provide a cost-effective alternative for improving health care delivery [10,11], with recent advances being made in digital primary care triage applications [12,13]. Using a digital triage tool has the potential to identify patients requiring emergency or urgent care in addition to supporting the planned allocation of appointments to better use the clinician skill-mix and improve clinician satisfaction [14]. In response to these points, measures of efficiency, cost savings, and clinician satisfaction were included as study secondary measures.

### Overview of the Digital Assessment Routing Tool

The Digital Assessment Routing Tool (DART) is a case-based reasoning digital triage system directing self-assessing patients with MSK conditions to the correct level of care, classified as a tier C system by the UK National Institute for Health and Care Excellence, with stratification configured to match the provider’s clinical services. DART uses the same-day emergency care principles described by NHS England, routing patients to the most appropriate care—right person, place, and time [15]. Previous work evaluating the safety and effectiveness of DART has been completed with promising results [14,16]. Given the potential for DART to support service delivery across an NHS MSK pathway, we embarked on this quality improvement study. To date, no similar studies evaluating a digital triage system specific to MSK conditions in a real-world context have been published.

### Problem Description

The study was conducted in collaboration with an NHS MSK service based in the northwest of England. The existing referral process consisted of patients being referred for MSK care via an e-Referral by their primary care physician, or via a self-referral route where the patient completed a web-based form or contacted the service administration team by telephone. Patients were booked into the first available physiotherapist or MSK physician appointment by an administrator, with no clinical validation of urgency or complexity. A waiting list of 8 weeks or more from referral to assessment presented a safety issue for patients requiring urgent care. With no triage in place, onward referrals consisting of multiple appointments and delays occurred, increasing the risk of poor outcomes and inefficient use of scarce clinical resources. Operational inefficiencies were highlighted, and clinician satisfaction was reported as low, primarily due to unbalanced patient caseloads relating to case complexity and physiotherapist level of experience and expertise.

### Study Aim and Rationale

The specific aim of this project was to evaluate the impact of introducing DART as a first point of contact digital triage system across an NHS service MSK pathway. The primary measure was patient safety, defined as the avoidance of unintended or unexpected harm to people during the provision of health care [17]. Within the rapidly evolving digital health landscape, a primary concern is to ensure the introduction of digital systems does not cause or contribute to adverse events [18]. A previous noninferior randomized controlled trial (RCT) pilot indicated an acceptable level of routing outcome agreement between DART and the physiotherapist, which provided the target outcome for our measurement of safety in this study [19].

Secondary measures of efficiency; cost-saving; and patient, clinician, and administrator satisfaction were selected following discussions with the NHS service leads, who highlighted these as factors important to their service. In addition to providing outcomes, this report describes the collaborative approach used in delivering the project in a live clinical service.

## Methods

### Context

The service receives an average of 1200 self-referrals per month. It is staffed by 39 physiotherapists of varying levels of experience, as defined by Agenda for Change banding [20], including Consultant Physiotherapy Practitioners and Advanced Clinical Practitioner Physiotherapists who provide clinical support to less experienced band 5-7 physiotherapists. An MSK physician provides a point of escalation in addition to delivering ultrasound-guided injections, minor surgery, and prescribing medications. The service is supported by 14 administrative staff. From the outset, key principles fundamental to the successful delivery of the project within a live clinical environment were acknowledged. These included no introduction of additional clinical risk or barriers to patients accessing care, and no increased burden on emergency services or primary care. Capacity was ensured to meet patients’ needs at every stage of the journey, and the impact on other parts of the pathway was considered.

### Study Design

We selected a Plan-Do-Study-Act (PDSA) cycle design, a methodology described and recommended for use by NHS England [21]. The PDSA cycle allows for iterative testing and learning within the complex and dynamic NHS environment, where changes can have unforeseen consequences. This approach reduces the risk of large-scale failures and ensures that improvements are evidence-based. The PDSA cycle’s flexibility ensures the intervention is fit-for-purpose and tailored to the specific context. However, it is acknowledged this can reduce the generalizability of results. To maximize the scientific rigor of the PDSA method, Taylor et al [22] emphasize the importance of following the design structure, both in application and reporting. This includes using lessons learned from one cycle to inform the next and using prediction-based expected or desirable levels of change, principles followed during our study [22,23].

Involving staff in the planning, execution, and evaluation of change fosters a sense of ownership and engagement. This not only improves morale but also ensures that knowledge and best practices are shared across the organization. To support this, we adopted an Integrated Knowledge Translation Approach between the NHS clinical team and researchers [24], not just in the planning stages but throughout the project, ensuring the methodology was relevant to a real-world NHS MSK pathway and connecting research to practice [25].

### Patient and Public Involvement

To assist with setting research priorities and outcome measures we invited 10 patients recruited at random from the service waiting area. Using a short questionnaire delivered by the lead researcher, we collected opinions on factors important to patients within the journey of their MSK condition (Multimedia Appendix 1). Most patients told us they were satisfied with their waiting time from first contact to the first appointment. One self-employed patient highlighted his loss of income during the time he was waiting because of his MSK condition. A total of 4 out of 10 patients were dissatisfied with multiple onward referrals between clinicians with no improvement in their condition. This information resulted in the reprioritization of our aim and measures, from measuring waiting time to measuring the effectiveness of DART stratification to direct the patients to the right type of intervention, the first time. Throughout the project, qualitative patient feedback was collected via a short questionnaire at the end of the DART assessment, informing iteration, and leading to integration of patient suggestions into the subsequent DART versions.

### Intervention Process

**Figure 1:**
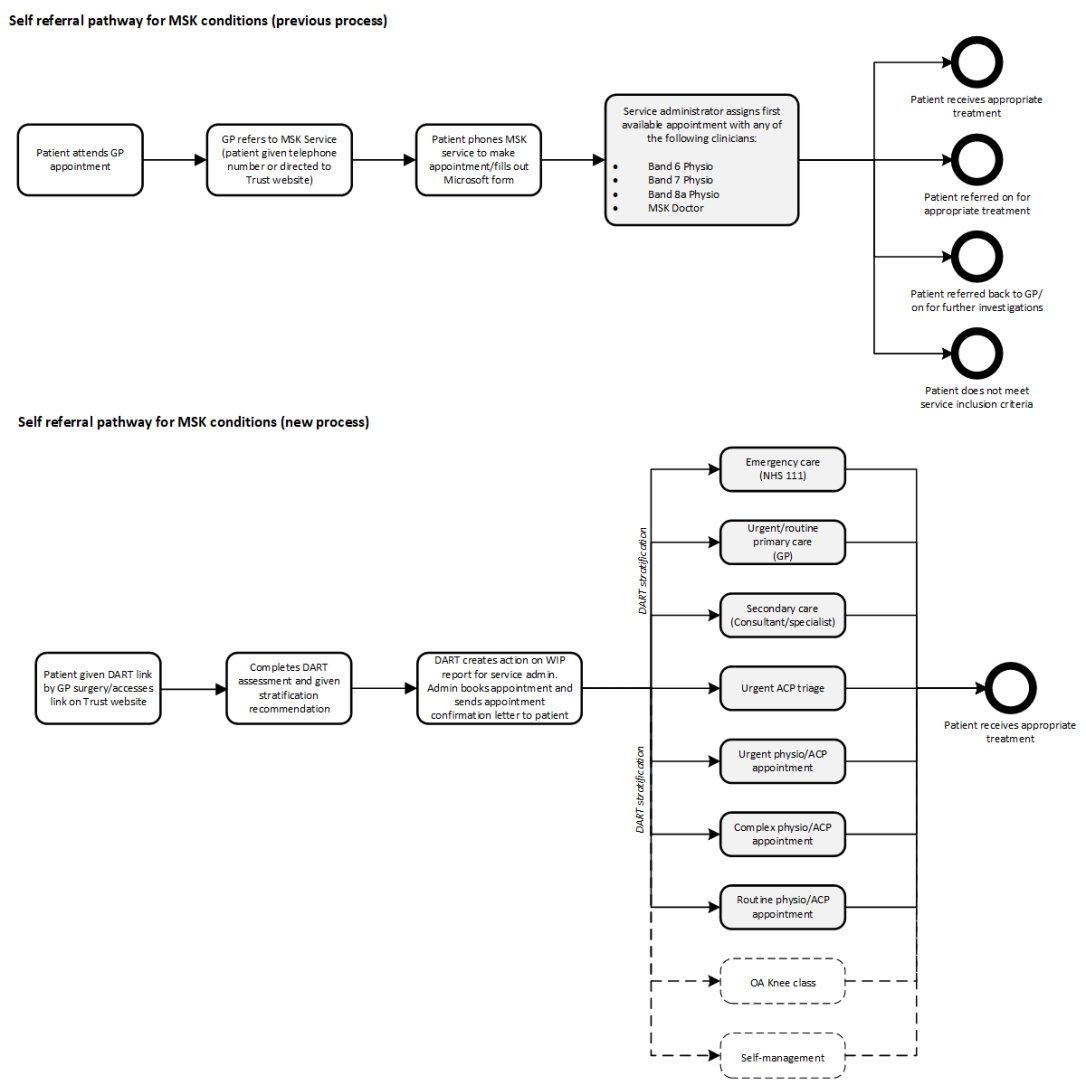
Self-referral quality improvement study of patients with musculoskeletal conditions, and previous and study intervention processes. This shows the introduction of DART triage stratification to newly created appointment options catering to the urgency and complexity of patient presentation, to support patients being directed to the right treatment, the first time. Outcomes within dotted lines indicate potential future service development informed by study data. ACP: Advanced Clinical Physiotherapist Practitioner; DART: digital assessment routing tool; GP: general practitioner; MSK: musculoskeletal; NHS: National Health Service.

DART was introduced as the first point of contact for all self-referring patients, replacing the existing web-based form or telephone self-referral process. Patients wanting to make a referral completed a DART assessment, accessed via the NHS service website, or delivered by an administrator over the telephone. The administrator booked an appointment corresponding to the DART stratification recommendation, with new types of appointments created to cater to urgency and complexity. In addition, DART collected data regarding the patient’s potential to self-manage their condition with which to inform future service development. The DART assessment clinical summary was uploaded to the NHS patient record by the administrator, allowing the receiving clinician to review it prior to the appointment. The existing primary care physician e-Referral process was retained to provide an alternative entry to the MSK pathway. Please refer to Figure 1 for existing and intervention processes.

### Measures

#### Primary Measure (Safety)

Safety was measured by the level of Clinician-DART stratification agreement, with the receiving clinician indicating their agreement or disagreement with DART stratification using a web-based form. Any disagreements were reviewed by the NHS service clinical leads to validate the result. A safety incident was defined as when a patient deemed by the clinician as requiring urgent assessment or treatment did not receive the necessary care in the appropriate timescales. Results from previous DART studies [19] and published work regarding generic symptom checkers [26] suggested an overall agreement level of 78% would be acceptable, but with no safety incidents. Measuring the number of patients identified by DART as requiring an urgent appointment provided an additional measure of safety, as in the existing process these patients would have remained on a waiting list for approximately 8 weeks without clinical review.

#### Secondary Measures

Secondary measures included efficiency, cost savings, and satisfaction, with all targets agreed jointly between the service lead and the researchers.

#### Efficiency

Efficiency measures were as follows:

1. The number of cases resulting in immediate onward referral between Band 6/7 and Band 8a physiotherapists to reduce (by any amount).
2. Time for administrators to process self-referrals, with a target of a 50% reduction across self-referrals.

#### Cost Savings

Cost-saving measures were as follows:

1. Administrative cost to process self-referrals, with a target of a 50% reduction across self-referrals.
2. Potential cost savings of patients being stratified to a physiotherapist-led remote self-management pathway, with a target of 50% reduction of cost per patient.

#### Satisfaction

A target was set at 84% of patients rating DART as “excellent, very good, good” or “fair.” This score was chosen as it would be comparable with previous DART usability studies and pilot RCT results [15,27]. Administrator satisfaction was measured qualitatively during and at the end of the project using web-based questionnaires, with an overall rating of “better” or the “same” as the existing process deemed to be acceptable. Clinician feedback was gathered but was not associated with a predefined target.

Performance related to these targets would ultimately guide decisions to recommend implementation, revision, or abandonment of DART’s inclusion within the MSK service.

#### Data Collection

Training was provided to both clinicians and administrators prior to starting data collection in the form of on-site visits, with researchers available for the first 2 days of data collection to monitor and assist with the new process as required.

#### Quantitative Data

Quantitative data were collected within the DART system, amalgamated and anonymized for clinical and service trend analysis, and exported in the form of spreadsheets by an Optima Health administrator. This included patient demographics, number of assessments, body site selection, stratification data, and time to complete assessment. Physios were asked to complete a short web-based form to register their level of agreement with the DART routing for each case.

It was intended that self-referral data for the 4 months prior to commencement of the study would be used for comparison, exported by the NHS service lead from the NHS record system. However, it transpired not all data fields were available for the study measures, and what was present was often found to be incomplete or inaccurate. This was a consideration during data analysis and is noted as a study limitation.

#### Qualitative Data

Patient satisfaction data were collected throughout the project via a short questionnaire and free text options embedded into the end of the DART assessment (Multimedia Appendix 2). This was anonymized and exported into a spreadsheet for analysis. Using unstructured group and individual meetings, clinicians gave feedback on their experience of the new process to the lead researcher, which was collated and documented (Multimedia Appendix 3). Administrators completed a web-based form to provide their opinions about the new self-referral process using DART (Multimedia Appendix 4). In addition, referring primary care physicians were invited to give feedback using a web-based survey, allowing assessment of the positive and negative impacts of DART introduction on their practice (Multimedia Appendix 5).

#### Sample Size

From previous DART development work, it was known that approximately 450 DART assessments would be sufficient to allow analysis of stratification trends. With service referral volumes averaging 300 per week, the first cycle was planned to last 2 weeks to allow analysis and confirmation of correct stratification and identify any safety critical changes required. The following 2 longer cycles provided time for process and DART system changes in response to data analysis, and to conclude if the DART intervention required amendment, adoption, or abandonment.

#### Data Analysis

All data were analyzed jointly between the lead researcher and the NHS service lead. The lead researcher is a qualified physiotherapist with a Master degree in MSK physiotherapy and is a final year PhD student at Queen Mary University of London. The NHS service lead is a Consultant Physiotherapist with 14 years of managing MSK services and a Regional Representative for the NHS England “Get It Right First Time” program. Weekly calls were scheduled to discuss project progress, in addition to an open channel of communication between researchers and personnel on-site to address any concerns or suggestions. As the new DART self-referral process completely replaced the previous process, any changes observed were considered due to the intervention, thus improving the validity and reliability of results.

### Ethical Considerations

It was confirmed by using the UK Medical Research Council decision tool that the study would not be considered as Research by NHS England. Therefore, neither Health Research Authority nor NHS Research Ethics Committee approval was required for the following reasons; participants were not randomized to different groups, the study protocol did not demand changing patient care from accepted standards for any of the patients involved and the study findings were specific to the study service and not generalizable. The outcome of the decision tool can be taken as authoritative. While there are currently no published ethical standards published for quality improvement studies, the guidance published by Hunt 2021 was followed [28]. In addition, the study protocol was reviewed and approved by the clinical governance and research and development offices of the NHS organization at which the project was conducted. Patients consented to the use of DART as part of the service’s new self-referral process or alternatively were signposted to an existing physician e-Referral route. Individual patients were not recruited into the study. All data collection related to the performance of the DART system itself and did not measure clinical patient outcomes or individual characteristics. Patients were provided access to the DART Privacy and Data Protection Policies prior to starting an assessment, which included descriptions of how their anonymized personal data would be used for purposes of managing their referral. The NHS service did not receive any remuneration for participating in the project and patients were not offered any financial reward to participate.

## Results

### Overview

During the 4-month project period (February-May 2024), the service received 4076 self-referrals, with all patients completing a DART assessment. This compared with 3818 referrals in the 4 months prior to the project, less because of the Christmas period. Patient demographics are shown in Table 1.

Patient ages ranged between 16 and 104 (mean 59) years, with 53% (n=2148) being 60 years or older. There was a higher percentage of females self-referring than males (2523/4076, 62% females, 1553/4076, 38% males), with the ratio being higher than commonly encountered in primary care [29].

Referrals by body site were largely comparable with those seen in primary care, with the exception of knee presentations (899/4076, 22%) being more prevalent than lower back (769/4076, 19%) [29]. On further analysis, 12% (n=106) of all patient knee presentations were assessed by DART as having been diagnosed or having symptoms of, osteoarthritis, likely explained by the high percentage of older patients.

As the study intervention process completely replaced the existing self-referral process, each cycle had to run concurrently with no break between cycles, ensuring patients were always able to access care. Data were collected and analyzed on a weekly basis throughout the study, with improvements for the next cycle discussed and agreed collaboratively between the researchers and the NHS clinical team. The next cycle commenced when the new DART iteration was deployed overnight with minimal break in DART accessibility (Table 2).

**Table 1:** Study patient demographics by age, presenting body site, and sex at birth (n=4076).

Category		Values
**Age (years), n (%)**		
	16-19	54 (1)
	20-29	204 (5)
	30-39	347 (9)
	40-49	455 (11)
	50-59	868 (21)
	60-69	1004 (25)
	70-79	799 (20)
	80+	345 (9)
Age (years), mean (range)		59 (16-104)
**Body site, n (%)**		
	Head and neck	243 (6)
	Chest and upper back	114 (3)
	Lower back and pelvis	769 (19)
	Shoulder	620 (15)
	Elbow	101 (2)
	Wrist and hand	370 (9)
	Hip	519 (13)
	Knee	899 (22)
	Foot and ankle	441 (11)
**Sex at birth, n (%)**		
	Male	1553 (38)
	Female	2523 (62)

**Table 2:** Table showing the duration of each Plan-Do-Study-Act (PDSA) cycle, areas identified within that cycle for improvement, source of data, and corresponding improvements made prior to the next testing cycle.

PDSA Cycle	Areas identified for improvement (data source)	Improvements included in next iteration
Cycle 1: Week 1-2 (2 weeks)	Patient feedback: non-DART^a^ access options, timelines for contact after assessment Research team: overview of referral process Administrators: delivery of assessment by telephone, safe management of referrals Service manager: increase DART process awareness to primary care physicians	Inclusion of service contact details within DART, contact time text added to outcome page Improved process information on preassessment screen Removal of sensitive clinical information for administrator user (escalation to physio), daily DART assessment summary sent to ensure no missed referrals by administrators Additional communications sent to physicians to encourage the use of DART self-referral process
Cycle 2: Week 3-10 (8 weeks)	Patient feedback: option to add additional comments Research team: DART algorithm refinement to safely reduce false positives for serious pathology, direct off-work patients to urgent appointments Administrators: hesitance of patients to complete DART themselves Service manager: add support for patients waiting for an appointment	Addition of free-text boxes on every DART assessment page Algorithm updates, creation of urgent appointment outcomes for off-work patients, and corresponding appointment type Change to administrator scripting and training in directing patients to DART self-assessment where possible Link to Digital Therapeutics online self-management content for patients waiting for routine appointments
Cycle 3 Week 11-16 (6 weeks)	Patient feedback: concerns about others not being able to access DART Research team: addition of free text box on every assessment page resulting in increased time to complete self-assessment, minor algorithm changes Administrators: ability to generate daily work-in-progress report Service manager: identification of high volume of knee osteoarthritis referrals resulting in the opportunity to create new stratification outcomes for condition-specific intervention	Preassessment content added to encourage seeking help from family or friends, telephone assessment, or GP^b^ referral Patients directed to one free box at the end of the assessment, and minor algorithm changes made To be delivered on completion of study subject to DART remaining in situ New routing outcome created stratifying patients to new intervention

^a^DART: digital assessment routing tool.

^b^GP: general practitioner.

It was imperative any safety or major operational issues were quickly identified and corrected and for this reason, the first PDSA cycle was deliberately short (2-week duration) to prevent service disruption. Clinician-DART agreement was not analyzed during this cycle due to insufficient volumes, but clinicians provided feedback via the web-based form regarding any routing they felt was not correct. Data from Cycle 1 informed changes to patient-facing wording to improve user engagement and confidence in DART and to address some small operational process changes.

Cycle 2 (weeks 3-10) provided a sufficient volume of DART assessments to gauge the level of stratification agreement and highlight DART algorithm changes requiring improvement in order to optimize routing to specific service requirements. Minor changes were made to the DART clinical algorithm to improve the accuracy of stratification and reduction of false positive routing. While DART identified patients absent from work or on reduced or adjusted duties, there was no appointment urgency associated with this routing outcome. It was suggested by the clinical team we introduce prioritization of appointments for absent patients to be seen within 2 weeks, thereby supporting the National Institute for Health and Care Excellence’s early intervention for workplace health guidelines [30], and this change was included for Cycle 3. Information was added to all nonurgent DART patient-facing outcome pages providing a link to Digital Health Therapeutics [31], allowing waiting patients easy access to evidence-based clinical content. This was aimed at potentially reducing waiting lists and supporting the “Waiting Well” initiative [32].

Cycle 3 (week 11-16) data analysis assessed the effectiveness of the changes made in the last iteration, with all being confirmed as satisfactory, with the exception of the addition of the free text box on every DART assessment page. This had increased the time taken for a patient to complete an assessment and to counter this, patients were directed to a single text box on the final page. Data analysis also revealed a previously unrecognized high number of patients presenting with symptoms of knee osteoarthritis. The NHS clinical team requested an update to the DART algorithm allowing these patients to be routed to a condition-specific intervention, which would support a more targeted approach to management and reduce the cost of care for this patient cohort. The final iteration of DART was introduced into the service at the end of the study, following the confirmation that DART was to be retained.

Changes to 2 quantitative measures were tracked over the course of the study, Clinician-DART routing agreement (measuring safety) and patient satisfaction (to gauge potential adoption by target users), and these were compared with their predefined study targets. Agreement data were collected during cycle 1 but not analyzed due to insufficient case numbers during this short cycle. It was interesting to note that both measures were consistent across cycles, with a slight increase in patient satisfaction across the study. Both remained above the predefined targets throughout (Table 3).

The outcomes for all measures are shown below in Table 4, together with their associated predefined targets.

**Table 3:** Changes in clinician-DART (digital assessment routing tool) routing agreement and patient satisfaction across the 3 Plan-Do-Study-Act cycles, together with overall study means. Routing agreement was not measured for cycle 1.

	Cycle 1	Cycle 2	Cycle 3	Study mean
Clinician-DART agreement (% correct, arguably correct)	N/A^a^	95	98	96
Patient satisfaction (% excellent, very good, good, fair)	87	89	91	90

^a^Not available.

**Table 4:** Primary and secondary measures, operation definitions and data collection method, prestudy data, predefined targets, study outcomes, and additional information.

Measures		Operational definition (data collection method)	Prestudy data	Predefined target	Study outcome	Additional information
**Safety**						
	—^a^	Clinician-DART^b^-routing agreement (web-based form)	N/A^c^	78%	96%	Target based on the result of DART RCT^d^ pilot study (21)
	—	Number of safety incidents (NHS^e^ reporting process)	0	0	0	Defined as when a patient deemed by the clinician as requiring urgent assessment or treatment did not receive the necessary care in the appropriate timescales
	—	Number of patients identified as requiring an urgent appointment	0	N/A	401	No triage in the existing process
**Efficiency**						
	—	Cases resulting in immediate onward referral at first appointment (NHS record system)	210	Reduction from previous prestudy period	Reduction by 203 cases	High complexity cases routed directly to Band 8a physiotherapist or MSK^f^ physician
	—	Time taken (mins) to process 100 self-referrals (NHS team and DART)	650	50% reduction across study	317 51% reduction	Attributable to 93% of patients completing DART self-assessment via web
**Cost savings**						
	—	Administrative cost to process 100 self-referrals (NHS team)	£156 (US $197.14)	50% reduction	£75.84 (US $95.84) 51% reduction Cost saving of £80.16 (US $101.30)	Reduction in cost related to reduced administrator time required to process self-referrals
	Existing process standard course of physiotherapy clinical costs per referral: £115.88 (US $146.44) (3.5 hours)	DART routing to physiotherapist-led self-management pathway (DART and NHS team)	Cost of 15% of 100 patients receiving standard course of physiotherapy £1738.20 (US $2196.56)	50% reduction in cost	Cost of 15% of 100 patients receiving self-management £465.30 (US $588) 73% reduction Cost saving of £1272.90 (US $1608.56)	Study showed 15% (611) of patients were routed to self-management Clinical costs per referral for new routing: (1 hour) £31.02 (US $39.11)
	—	DART routing to specific knee osteoarthritis program (DART and NHS team)	Cost of 2.6% of 100 patients receiving standard course of physiotherapy £347.64 (US $439.31)	50% reduction in cost	Cost of 2.6% of 100 patients receiving knee program £127.29 (US $160.86) 63% reduction Cost saving of £220.35 (US $278.46) saving	Study showed 2.6% (105) of patients could potentially be routed to knee program Clinical costs per referral for new routing: (1.25 hours) £42.43 (US $53.49)
**Satisfaction**						
	—	Patient satisfaction (rating scale and free text option at the end of DART assessment)	N/A	84% rating DART as “excellent, very good, good” or “fair”	90% based on a completion rate of 45.2%	“Good system saves time and enables appointment promptly.” [65-year-old male] “I'd rather see someone in person.” [45-year-old male]
	—	Clinician satisfaction (measured qualitatively using individual and group meetings)	N/A	Overall positive feedback	Overall positive feedback	“[DART summary] helps to shape questioning and can raise points that historically patients forget to divulge as part of a subjective [assessment]”. [Band 8a Physiotherapist] “DART routing can be influenced by patients over-exaggerating symptoms”. [Band 7 Physiotherapist]
	—	Administrator satisfaction (n=14) (measured using web-based questionnaires)	N/A	Overall rating of “better” or the “same” as existing process	DART process rated “better” or “same” by all administrators	“Most patients happy to go away and complete. Only small amount needing to be done over phone.” [administrator] “Doing a DART telephone assessment sometimes takes a long time with some patients.” [administrator]

^a^Not applicable.

^b^DART: digital assessment routing tool.

^c^N/A: data not reported in the existing process.

^d^RCT: randomized controlled trial.

^e^NHS: National Health Service.

^f^MSK: musculoskeletal.

### Primary Measure (Safety)

Across all study referrals, there was 96% Clinician-DART agreement, over the predefined limit of 78%, with no safety incidents reported. DART identified 401 (9.8%) patients as requiring an urgent appointment and stratified to a newly created urgent appointment to be seen within 2 weeks. In the existing process, these patients would have remained on a waiting list for approximately 8 weeks without clinical review.

### Secondary Measures

#### Efficiency

The number of cases requiring immediate onward referral between clinicians was reduced by 203 compared with the prestudy period, largely due to patients with more complex or potentially serious conditions being directed first time to more experienced clinicians with greater knowledge, skills, and competencies. Consequently, the amount of support required to support less experienced clinicians was reduced. This was confirmed during the poststudy meetings:

There has been a decreased level of clinical queries around complex pathology presentations from lower banded team members.

Band 8a Physiotherapist

Clinicians also reported more effective use of their allocated assessment time, due to having access to the DART clinical summary prior to the patient appointment.

It helps with assessment planning as I get more information before I see the patient.

Band 6 Physiotherapist

Previously, patient access to self-referral was limited to the administration team working hours of 7:30 AM to 5 PM, which increased to 24/7 using DART. All administrators answered “yes” when asked if their time was being used more effectively.

Great that the patients are positive about us sending them the email link and they can do it in their own time, and it means that we can get on to the next call so it’s quicker to answer the phone to the next caller.

administrator

The increased administrator time per call to deliver a DART assessment by telephone (9.5 minutes versus the existing process of 4 minutes) was offset by 93% of assessments being completed by patients self-assessing via the web, meaning an overall reduction in administrative time of 51%. This allowed administrators more time to assist patients requiring support and to complete other patient-facing activities. Some administrators told us it took them a long time to complete a DART assessment with the patient over the telephone, although this was not consistent across the team. It was suspected this was related to the individual administrator’s confidence in delivering the assessment, a factor that should be considered when delivering training to ensure consistency of delivery.

#### Cost Savings

For cost modeling purposes, cost savings associated with a reduction in administrator time and clinical time were calculated based on 100 referrals. The total cost to process a self-referral consisted of administrator-patient call time and the time taken to book an appointment and send a confirmation message to the patient, with an hourly administrator cost of £14.30 (US $18.07). There was a 51% cost saving equating to £80.16 (US $101.30) per 100 referrals.

DART identified 15% (n=611) of all patients being suitable for remote delivery of physiotherapist-led self-management with safety-netting and patient-initiated follow-up. Compared with the cost of the existing model of one face-to-face assessment and 3 treatments, this would represent a cost saving of 73% and £1272.90 (US $1608.56) across an intake of 100 referrals. However, further validation of self-management stratification percentage is recommended to ensure good outcomes are being achieved and patients not re-entering the pathway with the same condition.

During the study, DART identified 105 (2.6%) patients presenting with a knee problem that had been diagnosed with (or presented with symptoms of) mild to moderate knee osteoarthritis, and were potentially suitable for a tailored knee program as an alternative to a standard course of physiotherapy. This would represent a cost saving of 63% for this patient cohort and a potential £220.35 (US $278.46) per 100 referrals.

### Satisfaction

#### Patients

A total of 84% of patients rated DART as “excellent, very good, good” or “fair.” Qualitative feedback collected throughout the survey was largely positive, with key themes being quick and easy to use, and a good way to get an appointment. Negative comments were less frequent and mainly concerned with difficulty responding to specific questions and the preference to speak to a person about their problem. In response, the option for a patient to speak to an administrator to complete DART was reinforced on the service webpage next to the DART assessment link, and patients were asked to contact their primary care physician to make a referral if neither DART options were possible nor desirable.

#### Clinicians

No concerns were raised by clinicians around their job security or being “replaced by technology,” in fact, a key theme was the improved balance of urgent and complex presentations in their diaries resulting in better job satisfaction and well-being, supporting the NHS service Working Well strategy [33]:

There has been a significant improvement in team morale with better diary organisation and more structure for less experienced bands.

MSK Doctor

Possible disadvantages of DART mentioned were the quality of data entered by the patient, lack of detail provided by DART in more complex presentations, and the challenge of catering for multiple body site presentations. The potential for overreliance on the DART stratification during a clinical assessment was also raised. This could be a potential safety issue should the clinician fail to complete a thorough assessment of the patient. This was not encountered during the study, but it emphasizes the importance of thorough clinician training during DART implementation on how to safely and effectively use the DART clinical summary.

#### Administrators

While overall job satisfaction levels remained static, all administrators (n=14) rated the DART process as good as or better than the previous self-referral process. No administrators considered the process to be worse.

#### Primary Care Physicians

Physician practices were invited to provide feedback regarding the new process, which also served to improve engagement around the MSK service generally, with some practices not being aware patients were able to self-refer. Around 56% of self-referring patients had been given a link to DART by their practice, with anecdotal feedback indicating the new self-referral process enabled clinics to direct patients straight to DART instead of booking a physician appointment. Additionally, the study supported the promotion of service quality improvement initiatives within the service and across the wider NHS region.

### Unintended Benefit

Annual service funding is calculated by patient activity, with patients subject to exclusion criteria listed on the service website. DART identified 306 patients as ineligible to use the service and redirected them accordingly. This was an unintended outcome but would represent a cost saving of £28,476 (US $37,320)/annum to the service based on the cost of one initial assessment per case.

### The Project Overall

Initial deployment of DART into the MSK pathway was hampered by gaining access to the NHS internet and small operational issues, addressed within the iterative process, with no disruption to the service. No additional costs were incurred outside of the budget. Feedback regarding the quality improvement project overall was overwhelmingly positive and supported the collaborative design.

Being clinically led, it just worked.

Band 7 Physiotherapist

It was really well organised and not disruptive to the service.

Band 8a Physiotherapist

## Discussion

### Principal Findings

The aims of this project were achieved. The predefined targets for the primary outcome measure of safety were met. All of the secondary measures of efficiency, cost, and satisfaction were also achieved. The results demonstrated the benefits of adopting DART as the first point of contact in the NHS England MSK self-referral pathway.

Patient safety should always be the prime concern when implementing new digital health technology such as a digital triage (also known as symptom checkers) and is commonly measured by the level of agreement between the system and a clinical comparator. There is limited published evidence relating to the safety of digital triage with reported accuracy of routing varying considerably between 17% and 98% [12,34-38]. However, it is not possible to determine if this variation corresponds to true measures of system performance or is due to variability in testing methods. While there is no regulatory standard as to what constitutes clinical equivalence for digital triage, the 78% achieved by DART in our previous pilot RCT, with no clinical incidents, provided a predefined target for our measure of safety [19]. Our study agreement level of 90% far exceeded this, and it is of interest to reflect on why this could be the case. First, the quality improvement study was not subject to the controlled environment of the RCT pilot. Second, we introduced “arguably correct” as a routing agreement option for the receiving clinician, to account for safe and effective warranted clinical variation as described by Sutherland and Lavesque [39]. Thirdly, learning from the RCT pilot, thorough preparation work was completed with the NHS clinical team to tailor the DART algorithm to their specific service, which supported improved alignment of DART routing with the clinical services available. The level of safety was assessed by the NHS clinical team to be sufficient to adopt DART as the first point of contact within their MSK pathway and its use was continued following the conclusion of the quality improvement study.

Accessibility is often quoted as a barrier to digital health adoption [40,41], however, this study demonstrated that with the provision of alternative methods of DART delivery, most patients could complete a DART assessment. Encouraging patients to seek help from family or friends, using “surrogate seeking” to complete an assessment is a recognized way for people with lower levels of digital health literacy or nonnative English speakers to use a web-based system [42], a strategy previously reported in our DART usability study [27]. High levels of patient satisfaction, combined with no significant reduction in self-referral rates, led to the conclusion DART was not a significant barrier to self-referring patients accessing care.

The implementation of DART was well-received by less experienced physiotherapists, having a profound effect on their levels of work satisfaction. They told us they previously found the number of complex patients they saw a day challenging, and in some cases, “overwhelming.” Studies have shown the clinical decision-making skills required to assess complex presentations are acquired with the experience developed over several years post qualification and associated with more experienced higher-band physiotherapists [43]. Configuration of DART to stratify complex patients to specific bands of physiotherapists, supported a better balance of complex and simple presentations for less experienced clinicians, leading to improved work satisfaction, and additionally may aid retention of scarce clinical resources and reduce associated recruitment costs.

A key strength of the project was the collaborative approach, where all stakeholders remained engaged with data analysis and iteration from beginning to end. In addition, this proved instrumental in successfully embedding DART within the MSK pathway, overcoming the concerns of clinical and administrative teams often associated with deploying digital health technology [44,45]. Variations in NHS MSK services across England include referral routes, service exclusion criteria, clinical interventions available, and onward referral options, adding to the complexity of delivering an equitable service to patients. When introducing a digital triage system inevitably there is a trade-off between improving consistency across different NHS regions and matching stratification to the specific needs of the service. This can only be achieved by collaborative working between system developers and clinical service providers and this approach certainly supported the successful delivery of this project.

### Relationship With Existing Work

While primary care digital triage systems are increasingly suggested as an alternative to clinician-led triage, there remains scant evidence of their effectiveness. Between 2019 and 2023 alone there have been 6 literature or scoping reviews evaluating published studies of their performance [26,46-50], with the same conclusions being drawn that there remains no confirmation of their assumed potential benefits. Significant ontological, epistemological, and methodological limitations have been consistently identified, affecting the quality of most digital triage studies [51]. A tendency for developer bias is also well documented, likely linked to the cost and time to evaluate a system in a fast-paced competitive digital health market [47]. This has led to calls for a more rigorous independent evaluation process and greater market regulation [52]. In their scoping review published in 2019, Aboueid et al [46] went as far as to say that a prominent knowledge gap exists in this field, with further reviews having limited value until more high-quality research is available. Our quality improvement study was the culmination of a program of work to design robust study protocols and deliver the standard of research required to give reassurance to clinical service managers and commissioners.

### Limitations

While data collection methods and types were agreed upon during the study planning stage, it became apparent once the study had commenced that analysis and insight into the service were challenged by the availability and accuracy of data from the NHS electronic record system. This was due to a lack of key fields associated with study measures and user input errors associated with the complexity of the system’s data field format. A more detailed analysis would have been possible had this not been the case. Unlike a more formal form of research design, our quality improvement study had no direct comparator, which related to the pragmatic nature of the study in the time available. This project was conducted at one site with DART tailored to the specific local context, meaning the results are not generalizable, with NHS service differences, geographical factors, and patient demographics being key variables [47].

### Bias

Evaluation of digital health technology is commonly performed by the company who have developed the system, with research carrying an inherent risk of bias. This risk was present for our study, as Optima Health (the developer of DART) funded this research. The lead researcher is an employee of Optima Health, as well as a PhD student at Queen Mary University of London. Two other Optima Health employees assisted with the deployment of DART and training of administrators, however, were not involved in data analysis. To minimize bias, all quantitative data and patient qualitative data were collected within the DART or NHS electronic record system and analyzed in conjunction with the NHS service lead. A review of cases to assess safety and level of Clinician-DART agreement was completed by the NHS clinical team, with no researcher input. Clinician feedback was collected jointly by the lead researcher and NHS service clinical lead. Academic oversight and review of the study were provided by Queen Mary University of London, with the lead researcher having completed the National Institute of Health Research Good Clinical Practice and Standards in Research training course. The NHS service did not receive any remuneration for participating in the project. Patients were not offered any financial reward to participate in the study.

### Conclusions

This quality improvement study demonstrated the introduction of DART as the first point of contact in an MSK self-referral pathway produced positive outcomes in all measures, presenting opportunities to improve safety, efficiency, cost, and satisfaction across the study NHS service. While the results of the study are not directly transferable to other services, they do support the potential for improvement across other sites. In addition, the successful delivery of a quality improvement project using an Integrated Knowledge Translation Approach could provide a model for other researchers wishing to test and implement digital health within clinical services.

## Appendix

Multimedia Appendix 1 Prestudy patient questionnaire.

Multimedia Appendix 2 Patient satisfaction questionnaire completed at the end of the DART assessment.

Multimedia Appendix 3 Poststudy clinician questionnaire.

Multimedia Appendix 4 Administrator post-study survey.

Multimedia Appendix 5 Poststudy primary care physician survey.

## Abbreviations

DART: digital assessment routing tool
MSK: musculoskeletal
NHS: National Health Service
PDSA: Plan-Do-Study-Act
RCT: randomized controlled trial
SQUIRE: Standards for Quality Improvement Reporting Excellence Framework

## Data Availability

The datasets generated or analyzed during this study are not publicly available due to them being stored within the DART system, but are available from the corresponding author on reasonable request.
