# Supplementary material for: Improving Safety, Efficiency, Cost, and Satisfaction Across a Musculoskeletal Pathway Using the Digital Assessment Routing Tool for Triage: Quality Improvement Study
Source: J Med Internet Res. 2025 Apr 25;27:e67269. doi: 10.2196/67269 (PMC12064960; doi:10.2196/67269)
Supplement: Multimedia Appendix 1 [file jmir_v27i1e67269_app1.pdf]

NHS
Mersey and West Lancashire Teaching Hospitals
NHS Trust

Physiotherapy service patient questionnaire

How did you access the physio service?

☐ My GP referred me

☐ I referred myself

How did you find that process?

E.g.<br><br>*Was it easy to find out how to do it?*<br>*Was it difficult to make the referral yourself?*<br>*Did you need help to do it?*<br>*How long did it take?*<br>*Did you understand what would happen next?*<br>*Did you know how long you would be likely to wait?*	

How long did you wait before you spoke to/saw a physio?

☐ Less than 2 weeks

☐ 2 – 6 weeks

☐ More than 6 weeks

Overall, how would you rate the current way of getting help for your problem?

☐ Very good

☐ Good

☐ Fair

☐ Poor

☐ Very poor

What do you think we could do to improve this referral process?
