# Supplementary material for: Improving Safety, Efficiency, Cost, and Satisfaction Across a Musculoskeletal Pathway Using the Digital Assessment Routing Tool for Triage: Quality Improvement Study
Source: J Med Internet Res. 2025 Apr 25;27:e67269. doi: 10.2196/67269 (PMC12064960; doi:10.2196/67269)
Supplement: Multimedia Appendix 2 [file jmir_v27i1e67269_app2.pdf]

NHS
Mersey and West Lancashire
Teaching Hospitals
NHS Trust

Mersey & West Lancashire Self-referral satisfaction survey

We would be very grateful if you could give us feedback on how you found this assessment process by answering these quick questions.

Please note this is feedback for the online assessment you have just done and should not be used for questions regarding appointments or symptoms. If you have any questions of this nature, please contact the Physiotherapy service on 01695 656268.

* Required

1. **How was this assessment completed?** *

◯ I completed it myself

◯ A friend or family member helped me

◯ A Joint Health Service administrator completed the assessment for me

2. **Having just completed your assessment, do you trust the system you used?** *

◯ Yes

◯ No

◯ Unable to say

3. **Are you going to act on the recommendation(s) you were given?** *

◯ Yes

◯ No

◯ Maybe

4. **Overall, how would you rate this way of getting help for your problem?** *

- ◯ Very good
- ◯ Good
- ◯ Fair
- ◯ Poor
- ◯ Very poor

5. **How did you find out about this assessment?** *

- ◯ My GP/GP surgery told me about it
- ◯ I called the Physiotherapy service and was sent the link via email
- ◯ I found the link on the hospital's website
- ◯ Other

6. **Do you have any other comments you would like to add?**

This content is neither created nor endorsed by Microsoft. The data you submit will be sent to the form owner.

Microsoft Forms
