# Supplementary material for: Improving Safety, Efficiency, Cost, and Satisfaction Across a Musculoskeletal Pathway Using the Digital Assessment Routing Tool for Triage: Quality Improvement Study
Source: J Med Internet Res. 2025 Apr 25;27:e67269. doi: 10.2196/67269 (PMC12064960; doi:10.2196/67269)
Supplement: Multimedia Appendix 3 [file jmir_v27i1e67269_app3.pdf]

Post-QIS clinician questionnaire

Clinician Banding:

How was your overall experience of using DART?

What benefits do you see for patients?

What about for your clinical practice?

What disadvantages do you think there may be?

Can you think of any areas of improvement, not just DART itself, but how it works within the MSK pathway?

Any final thoughts?
