# Supplementary material for: Improving Safety, Efficiency, Cost, and Satisfaction Across a Musculoskeletal Pathway Using the Digital Assessment Routing Tool for Triage: Quality Improvement Study
Source: J Med Internet Res. 2025 Apr 25;27:e67269. doi: 10.2196/67269 (PMC12064960; doi:10.2196/67269)
Supplement: Multimedia Appendix 4 [file jmir_v27i1e67269_app4.pdf]

Post-QIS administrator questionnaire

Before DART

How did you feel about the old self-referral process?	
• Do you think patients found it easy to self-refer?<br>• Do you think your time was being used effectively?<br>• Any other comments?	
**Overall, how would you rate the old self-referral process?**	
**5** - Very good<br>**4** - Good<br>**3** - Fair<br>**2** - Poor<br>**1** - Very poor	
**Overall, how satisfied were you with your job role?**	
**5** - Very satisfied<br>**4** – Somewhat satisfied<br>**3** – Neither satisfied nor dissatisfied<br>**2** - Somewhat dissatisfied<br>**1** - Very dissatisfied	

After DART

How do you feel about the new self-referral process?	
• Do you think patients find it easy to self-refer?<br>• Do you think your time is being used effectively?<br>• Any other comments?	
**Overall, how would you rate the new self-referral process?**	
**5** - Very good<br>**4** - Good<br>**3** - Fair<br>**2** - Poor<br>**1** - Very poor	
**Overall, how satisfied are you with your job role now?**	
**5** - Very satisfied<br>**4** – Somewhat satisfied<br>**3** – Neither satisfied nor dissatisfied<br>**2** - Somewhat dissatisfied<br>**1** - Very dissatisfied	

**Do you have any other comments you'd like to add?**
