# Supplementary material for: Improving Safety, Efficiency, Cost, and Satisfaction Across a Musculoskeletal Pathway Using the Digital Assessment Routing Tool for Triage: Quality Improvement Study
Source: J Med Internet Res. 2025 Apr 25;27:e67269. doi: 10.2196/67269 (PMC12064960; doi:10.2196/67269)
Supplement: Multimedia Appendix 5 [file jmir_v27i1e67269_app5.pdf]

Joint Health Musculoskeletal Interface Service
Digital Assessment Routing Tool (DART)

Please use this form to give us your feedback about the DART self-referral process implemented in January.

* Required

1. Was your practice aware of the self-referral process **before** DART was implemented? *

   ◯ Yes

   ◯ No

   ◯ Unsure

2. Was your practice made aware of the new process (DART) at the end of January? *

   ◯ Yes

   ◯ No

   ◯ Unsure

3. Did you make use of any of the marketing materials sent through at the time (and in subsequent emails)? *

   ◯ Yes

   ◯ No

   ◯ Unsure

4. Has the new musculoskeletal self-referral process enabled you to direct patients straight to DART (instead of booking patients into a GP appointment)?

   If so - would you say this has improved the process for patients and administrative/clinical staff? *

5. Do you have any other comments? *
